# Microsomal Triglyceride Transfer Protein (MTP) Associates with Cytosolic Lipid Droplets in 3T3-L1 Adipocytes

**DOI:** 10.1371/journal.pone.0135598

**Published:** 2015-08-12

**Authors:** Joseph D. Love, Takashi Suzuki, Delia B. Robinson, Carla M. Harris, Joyce E. Johnson, Peter J. Mohler, W. Gray Jerome, Larry L. Swift

**Affiliations:** 1 Department of Pathology, Microbiology and Immunology, Vanderbilt University School of Medicine, Nashville, Tennessee, United States of America; 2 The Ohio State University Wexner Medical Center, The Dorothy M. Davis Heart & Lung Research Institute and Department of Physiology and Cell Biology, Columbus, Ohio, United States of America; 3 Research Service, Veterans Affairs, Tennessee Valley Health Care System, Nashville, Tennessee, United States of America; Fundação Oswaldo Cruz, BRAZIL

## Abstract

Lipid droplets are intracellular energy storage organelles composed of a hydrophobic core of neutral lipid, surrounded by a monolayer of phospholipid and a diverse array of proteins. The function of the vast majority of these proteins with regard to the formation and/or turnover of lipid droplets is unknown. Our laboratory was the first to report that microsomal triglyceride transfer protein (MTP), a lipid transfer protein essential for the assembly of triglyceride-rich lipoproteins, was expressed in adipose tissue of humans and mice. In addition, our studies suggested that MTP was associated with lipid droplets in both brown and white fat. Our observations led us to hypothesize that MTP plays a key role in lipid droplet formation and/or turnover. The objective of these studies was to gain insight into the function of MTP in adipocytes. Using molecular, biochemical, and morphologic approaches we have shown: 1) MTP protein levels increase nearly five-fold as 3T3-L1 cells differentiate into adipocytes. 2) As 3T3-L1 cells undergo differentiation, MTP moves from the juxtanuclear region of the cell to the surface of lipid droplets. MTP and perilipin 2, a major lipid droplet surface protein, are found on the same droplets; however, MTP does not co-localize with perilipin 2. 3) Inhibition of MTP activity has no effect on the movement of triglyceride out of the cell either as a lipid complex or via lipolysis. 4) MTP is found associated with lipid droplets within hepatocytes from human fatty livers, suggesting that association of MTP with lipid droplets is not restricted to adipocytes. In summary, our data demonstrate that MTP is a lipid droplet-associated protein. Its location on the surface of the droplet in adipocytes and hepatocytes, coupled with its known function as a lipid transfer protein and its increased expression during adipocyte differentiation suggest a role in lipid droplet biology.

## Introduction

Lipid droplets are intracellular energy storage organelles found in organisms as diverse as bacteria and mammals. They are composed of a hydrophobic core of neutral lipid (triglyceride and/or cholesteryl ester) surrounded by a monolayer of phospholipid and proteins. Lipid droplets were once thought to serve only as reservoirs for energy storage; however, more recent studies have revealed that droplets are not static, but are dynamic organelles that interact with other organelles, such as the endoplasmic reticulum (ER) and mitochondria [1, 2], and serve a variety of functions within the cell [3]. The dynamic nature of the droplet is reflected, in part, by the diverse array of proteins that have been identified to associate with the droplet. Major surface proteins include members of the perilipin family (previously termed the PAT family for perilipin, adipophilin, TIP47) [4]. This family encompasses five homologous proteins (perilipins 1–5) that have been shown to serve different roles in the genesis and turnover of droplets [4]. In addition to these well-studied proteins, proteomic studies have identified a number of other proteins associated with droplets in a variety of cells [5–16]. It is important to note that the proteins associated with the droplet are in many cases cell type-dependent, although there are certainly proteins common to most droplets. For example, proteins involved in lipid metabolism seem to be components of droplets in all cell types, as are proteins involved in intracellular traffic or signaling.

Clearly, the proteome of lipid droplets is extensive and expansive; however, the function of the vast majority of these proteins with regard to the formation and/or turnover of lipid droplets is unknown. Some of these proteins may not even have a function in the biology of the lipid droplet. Cermelli *et al*. have made a case that lipid droplets not only serve as storage depots for energy, but they may also serve as transient storage depots for proteins [5]. Understanding which proteins are simply transient “residents” and those that serve integral functions in the life of the lipid droplet is clearly important. Furthermore, although the list of proteins that have been identified with lipid droplets is long, it is not clear if all of the key proteins have been identified.

In this paper we demonstrate that microsomal triglyceride transfer protein (MTP), a lipid transfer protein with a high specificity for triglyceride and cholesteryl ester [17, 18], is associated with lipid droplets in adipocytes and hepatocytes. MTP is a heterodimeric protein complex consisting of a unique 97 kD protein with lipid transfer activity and protein disulfide isomerase (PDI). It has been shown to be essential for the assembly of triglyceride-rich, apolipoprotein (apo)B-containing lipoproteins by hepatocytes and enterocytes [19]. Our laboratory was the first to report that MTP is also expressed in adipocytes of mice, rats, and humans [20]. Immunohistochemical studies revealed MTP surrounding lipid droplets in both brown and white fat in mice. Whereas it was concentrated around the smaller droplets in brown fat and appeared to be associated with the surface, we could not discern if it was on the droplet surface in white fat because the large droplets tend to crowd other components into the remaining non-lipid areas of the cytoplasm. Immunofluorescence studies in 3T3-L1 adipocytes suggested that MTP was associated with the surface of the droplet and especially prominent on small (<5 μm diameter) lipid droplets [20]. In the current study we have utilized a series of microscopic and biochemical studies to probe more extensively the location of MTP with regard to the lipid droplet. In addition, we have investigated possible roles for MTP in lipid droplet biology. Our studies provide convincing evidence that MTP is a lipid droplet-associated protein. Based on the known function of this protein and its location with the droplet, one can easily envision a role for MTP in lipid droplet formation and/or maturation.

## Materials and Methods

### Antibodies and reagents

Rabbit anti-MTP was developed in our laboratory and has been described previously [21]. Goat anti-perilipin 2 was kindly provided by Dr. Carole Sztalryd Woodle (University of Maryland) and used for immunoblots. Chicken anti-perilipin 2 (AB 37516) was purchased from ABCAM (Cambridge, MA) and used for immunocytochemistry. Antibodies to GBF1 (SC-136240) and GRP78 (SC-376768) were mouse monoclonal antibodies purchased from Santa Cruz Biotechnology, Inc., Dallas, TX. Goat anti-rabbit IgG conjugated with Alexa 594 (AB 150080), goat anti-mouse IgG conjugated with Alexa 488 (AB 150113) and goat anti-chicken IgG conjugated with Alexa 488 (AB 150173) were purchased from ABCAM. BODIPY 493/503 was purchased from Life Technologies. Goat anti-rabbit IgG conjugated with 10 nm gold particles (25108) was purchased from Electron Microscopy Sciences (Hatfield, PA). MTP inhibitors BMS197636 and BMS212122 were kindly provided by R. Gregg, Bristol Myers Squibb, Princeton, NJ; CP346086 was purchased from Sigma-Aldrich Co., St. Louis, MO. Acetic acid, [1-^14^C], sodium salt (5.3 mC_i_/mmol)was purchased from MP Biomedicals, Santa Ana, CA. Isoproterenol hydrochloride (I6504) was purchased from Sigma-Aldrich.

### Cell culture

3T3-L1 cells were obtained from American Type Culture Collection (Manassas, VA) and were maintained in Dulbecco's modified Eagle's medium (DMEM) supplemented with 10% fetal bovine serum, 4 mM glutamine, 4500 mg/liter glucose, 1 mM sodium pyruvate, and 1500 mg/liter sodium bicarbonate. To maintain cells in a pre-differentiated state, they were not allowed to reach confluence. Differentiation of 3T3-L1 cells into adipocytes was initiated by the addition of 10 μM dexamethasone, 0.5 mM isobutylmethylxanthine, and 10 μg/ml insulin to confluent cells for 2 days. The cells were then cultured in medium containing 10 μg/ml insulin for 2 days after which they were maintained in complete media. The medium was changed every 2 days for 6 to 8 days, at which time differentiation was complete.

3T3-L1 cells were solubilized in 20 mM HEPES (pH 7.4), 1.0 mM EGTA, 1% Triton X-100, and 10% glycerol on ice for 20 min. The extracts were then centrifuged at 4°C for 5 min at 14,000 x *g* in an Eppendorf microfuge. The supernatant was recovered, and protein concentration was determined using the bicinchoninic acid (BCA) method (Thermo Fisher Scientific, Waltham, MA). Aliquots were taken for SDS-PAGE as described below.

### Triglyceride secretion from 3T3-L1 adipocytes

3T3-L1 cells were grown to confluence and induced to differentiate as described above. On day 6 of differentiation, the media was removed and serum-free media containing 2% fatty acid free bovine serum albumin (BSA) with or without MTP inhibitor (CP346086, 30 nM) was added [22]. The cells were incubated for 24 hr, at which time both media and cells were recovered. Lipids were extracted from media and cells, separated by thin layer chromatography and quantitated by gas chromatography as described below. Total protein in the cell fraction was determined using the bicinchoninic acid (BCA) assay.

Lipid secretion was also assessed using radioisotope labeling. Cells were grown in 6-well plates and differentiated. On day 6, 2 μC_i_ [^14^C]-acetate was added to each well and incubated for 24 hr. The media was removed, the cells washed and fresh media was added with and without CP346086 (30 nM). At 2, 4, 8, and 16 hr, a standard time frame for monitoring cellular lipoprotein secretion, the media and cells were collected. Lipids were extracted, separated by thin layer chromatography, and radioactivity determined by liquid scintillation counting.

### Lipolysis experiments

3T3-L1 cells were grown to confluence and induced to differentiate as described above. On day 8, MTP inhibitor (CP346086, 30 nM) or carrier (DMSO) was added to the cells for 45 min, after which the media was removed and replaced with fresh media (DMEM, 2% fatty acid free BSA) with or without inhibitor and with or without the β-adrenergic agonist isoproterenol (100 μM). Twenty minutes later the media was removed and fatty acids were extracted, purified by thin layer chromatography, methylated, and quantified by gas chromatography as described below.

### Immunocytochemistry

3T3-L1 cells were grown in 8-well chamber slides (Nunc Lab-Tek Chamber Slide System, Fisher Scientific, Norcross, GA) and induced to differentiate as described above. Cells were washed three times with phosphate buffered saline (PBS) and fixed with 2% paraformaldehyde for 30 min at room temperature. The cells were washed two times with intracellular buffer (75 mM potassium acetate, 2.5 mM magnesium acetate, 1.8 mM calcium chloride, 25 mM HEPES buffer, pH 7.2) at room temperature with a final rinse in 10% glycine in PBS. They were permeabilized with 0.1% saponin in intracellular buffer with 0.1% BSA for 30 min at room temperature. All subsequent steps, including antibody incubations and washes, were performed in intracellular buffer containing 0.1% saponin and 0.1% BSA. The cells were incubated overnight at 4°C with anti-MTP (1:500 dilution), washed three times for 10 min each, and incubated with an Alexa 594 conjugated secondary antibody for 2 hr at room temperature. After three washes of 10 min each, the cells were mounted with ProLong Gold Antifade (Molecular Probes, Eugene, OR). When two antibodies were involved, each primary and secondary antibody was added in succession. For example, in the case of staining for MTP and GBF1, anti-MTP was added followed by Alexa 594-conjugated secondary antibody. Anti-GBF1 was then added followed by Alexa 488 conjugated secondary antibody. Anti GBF1 was used at 1:250 to 1:400 dilutions; antiGRP78 was used at dilutions from 1:50 to 1:250; and anti perilipin 2 (ABCAM) was used at 1:400 dilution.

### Immunofluorescence microscopy

Wide-field fluorescence and DIC images were collected using a Zeiss Axioplan microscope (Zeiss, Oberkochen, Germany) equipped with a Photometrics Coolsnap HQ digital camera (Roper Scientific, Tucson, AZ). Confocal images were collected and analyzed on a Zeiss LSM 710 META Inverted laser scanning confocal microscope using Carl Zeiss ZEN 2012 imaging software and equipped with a Plan-Apochromat 63x/1.40 oil lens. Image slices of 0.9 μm were created (using Nyquist theorem) and arranged into an image stack. Parameters for imaging were set to capture the full fluorescence dynamic range without under- or over-saturation. Alexa 488 fluorescence was captured using a 493 dicroic and 488 long pass filter. Alexa 594 fluorescence was captured using a 587 dicroic and 561 long pass filter.

The overlap of MTP and perilipin 2 fluorescence was assessed from the confocal images. Under the conditions used to capture the images, the resolution was approximately 0.2 μm. Twenty five cells, which contained lipid droplets with MTP on the surface, were analyzed to determine if the fluorescence from MTP occurred within the same resolved space (~0.2 μm diameter) as the fluorescence from perilipin 2. Data were expressed as the percent of MTP fluorescence that overlapped with fluorescence from perilipin 2.

### Immunoelectron microscopy studies

3T3-L1 cells were induced to differentiate and on day 6, the cells were briefly fixed in 2% paraformaldehyde and embedded in LR white resin. Thin sections were cut and mounted on Formvar-coated grids. The grids were hydrated, and non-specific sites blocked with non-immune host serum prior to incubation with antibody to MTP and secondary antibody (goat anti-rabbit IgG) conjugated to colloidal gold (10 nm). Images were captured using a Philips/FEI CM-12 electron microscope.

### Isolation of lipid droplets

3T3-L1 cells were induced to differentiate, and on day 6 the cells were recovered and a lipid droplet fraction isolated according to Brasaemle and Wolins [23]. Briefly, the procedure was as follows: Cells were lysed in hypotonic lysis medium (HLM) using a Potter-Elvehjem tissue homogenizer, and nuclei, unbroken cells, and cell debris pelleted. The supernatant, containing the lipid droplets, was adjusted to 20% sucrose and overlayered with 5% sucrose, and HLM. Lipid droplets were floated to the top of the discontinuous sucrose gradient using a Beckman SW40 rotor (28,000 rpm, 30 min) and recovered by tube slicing. The lipid droplets were divided into three equal fractions. Fraction 1 (Control) was kept on ice. To fraction 2 (Washed) an equal volume of HLM was added; to fraction 3 (Hi pH) an equal volume of 0.2 M sodium carbonate, pH 11.0, was added. Both fractions were incubated on ice for 20 min. The density of fractions 2 and 3 was then adjusted to 20% sucrose, and lipid droplets were floated to the top of the discontinuous sucrose gradient using the SW40 rotor and recovered as before. All three fractions were then delipidated using -80°C acetone [23]. Triglycerides and phospholipids were separated by thin layer chromatography and quantitated by gas liquid chromatography. The precipitated proteins were solubilized in SDS PAGE solubilizing buffer and analyzed by immunoblotting.

### Immunohistochemistry

Paraffin-embedded sections of human liver were obtained through the Vanderbilt Translational Pathology Shared Resource. The paraffin was removed with xylene, and the tissues were equilibrated in tris-buffered saline (TBS). Antigen retrieval was performed by heating in 10 mM citrate (pH 6.0) for 10 min at 100°C. To block nonspecific binding, the slides were incubated for 1 hr at room temperature in TBS containing 3% (w/v) BSA and 1% normal goat serum. This blocking agent was also used to dilute the primary antibodies. Incubation with the affinity-purified MTP antibody (1:1000 dilution of the affinity purified IgG fraction, O.D. = 0.85) was carried out in a humidified chamber overnight at 4°C. The slides were then rinsed and incubated for 1 hr at room temperature with biotin-conjugated goat anti-rabbit IgG. The slides were rinsed and incubated at room temperature for 1 hr with anti-biotin antibody linked to alkaline phosphatase followed by incubation with alkaline phosphatase substrate for 20–30 min. Tissues were counterstained with hematoxylin and mounted with aqueous mounting medium (Serotec, Raleigh, NC).

### Reverse transcriptase—PCR

3T3-L1 cells were grown to confluence and induced to differentiate as described above. At days 0, 2, 4, 6, and 8, the cells were recovered, and total RNA was isolated using Trizol reagent (Life Technologies, Grand Island, NY) according to the manufacturer’s instruction. cDNAs were synthesized using Super Script III Reverse Transcriptase (Life Technologies) according to the manufactures instruction. In brief, 1 μg of total RNA was combined with random hexamers (Life Technologies) and oligo d(T)_16_ (Life Technologies) (both 1.25 μM final concentration) and dNTP mix (Sigma-Aldrich, 0.5 mM final concentration). The mixture was heated at 65°C for 5 min and then quickly chilled on ice for 5 min. 5X first strand reaction buffer, DTT (10 mM final concentration) and Super Script III enzyme (100 U) were then added, and the mixture was incubated at 25°C for 5 min, 50°C for 60 min, 70°C for 15 min. Semi-quantitative RT-PCR analysis was performed with primer sets for MTP-A (sense: AGTGCTTTTTCTCTGCTTCTTCTC, antisense: ATTTTGTAGCCCACGCTGTC, x35 cycles), MTP-B (sense: TGCCGTGCTGTTACTCTTTC, antisense: ATTTTGTAGCCCACGCTGTC, x28 cycles) and C/EBPα (sense: AAGCCAAGAAGTCGGTGGAC, antisense: CGGTCATTGTCACTGGTCAA, x28 cycles). For normalization, we used Non-POU-domain containing octamer binding protein (NoNo) (sense: TGCTCCTGTGCCACCTGGTACTC, antisense: CCGGAGCTGGACGGTTGAATGC, x22 cycles), which has been shown to be relatively stable during 3T3 cell differentiation [24]. PCR cycle conditions were 95°C for 30 sec, 60°C for 30 sec, 72°C for 30 sec with the indicated cycle numbers. Preliminary PCR experiments were run with MTP-A, MTP-B, C/EBPα, and NoNo using variable cycle numbers to determine optimal conditions for amplification.

### SDS-polyacrylamide gel electrophoresis and immunoblotting

Samples were solubilized in NuPAGE LDS sample buffer and separated by SDS-PAGE using NuPAGE bis-tris gels (4–12% gradients) (Life Technologies) with morpholinepropanesulfonic acid SDS running buffer [25]. The proteins were transferred to nitrocellulose membranes. The membranes were blocked in TBS with 5% non-fat milk, incubated overnight at 4°C with primary antibody, washed extensively, and incubated for 1 hr at room temperature with the appropriate secondary antibody conjugated with horseradish peroxidase. Bands were visualized using enhanced chemiluminescence (Amersham Pharmacia Biotech, Piscataway, NJ) and quantitated by densitometry (BioRad Model GS-700 Imaging Densitometer).

### Separation and quantitation of lipids

Lipids were extracted from cells using the method of Folch-Lees [26] and from lipid droplets using acetone [23]. Individual lipid classes were separated by thin layer chromatography using Silica Gel 60 A plates developed in petroleum ether, ethyl ether, acetic acid (80:20:1) and visualized by rhodamine 6G. Phospholipids and triglycerides, or unesterified fatty acids were scraped from the plates and methylated using BF3 /methanol (Sigma-Aldrich,St. Louis, MO) as described by Morrison and Smith [27]. The methylated fatty acids were extracted and analyzed by gas chromatography. Gas chromatographic analyses were carried out on an Agilent 7890A gas chromatograph equipped with flame ionization detectors and a capillary column (SP2380, 0.25 mm x 30 m, 0.25 μm film, Sigma-Aldrich). Helium was used as the carrier gas. The oven temperature was programmed from 160°C to 230°C at 4°C/min. Fatty acid methyl esters were identified by comparing the retention times to those of known standards. Inclusion of lipid standards (dipentadecanoyl phosphatidylcholine (C15:0), trieicosenoin (C20:1ω9), and pentadecanoic acid (C15:0)) permitted quantitation of the lipid classes.

### Statistical analyses

Statistical analysis was performed using Student’s *t* test or one-way analysis of variance (ANOVA) with Tukey’s *post hoc* test. Data are presented as the means ± S.D. p values < 0.05 were considered statistically significant.

### Ethics statement

De-identified human tissue slides were obtained from the Translational Pathology Shared Resource, Vanderbilt University Medical Center. The Institutional Review Board determined this study did not qualify as “human subject” research per §46.102(f)(2). Consequently, informed consent was not required (IRB# 141541).

## Results

### MTP expression in 3T3-L1 cells upon differentiation

**Fig 1:**
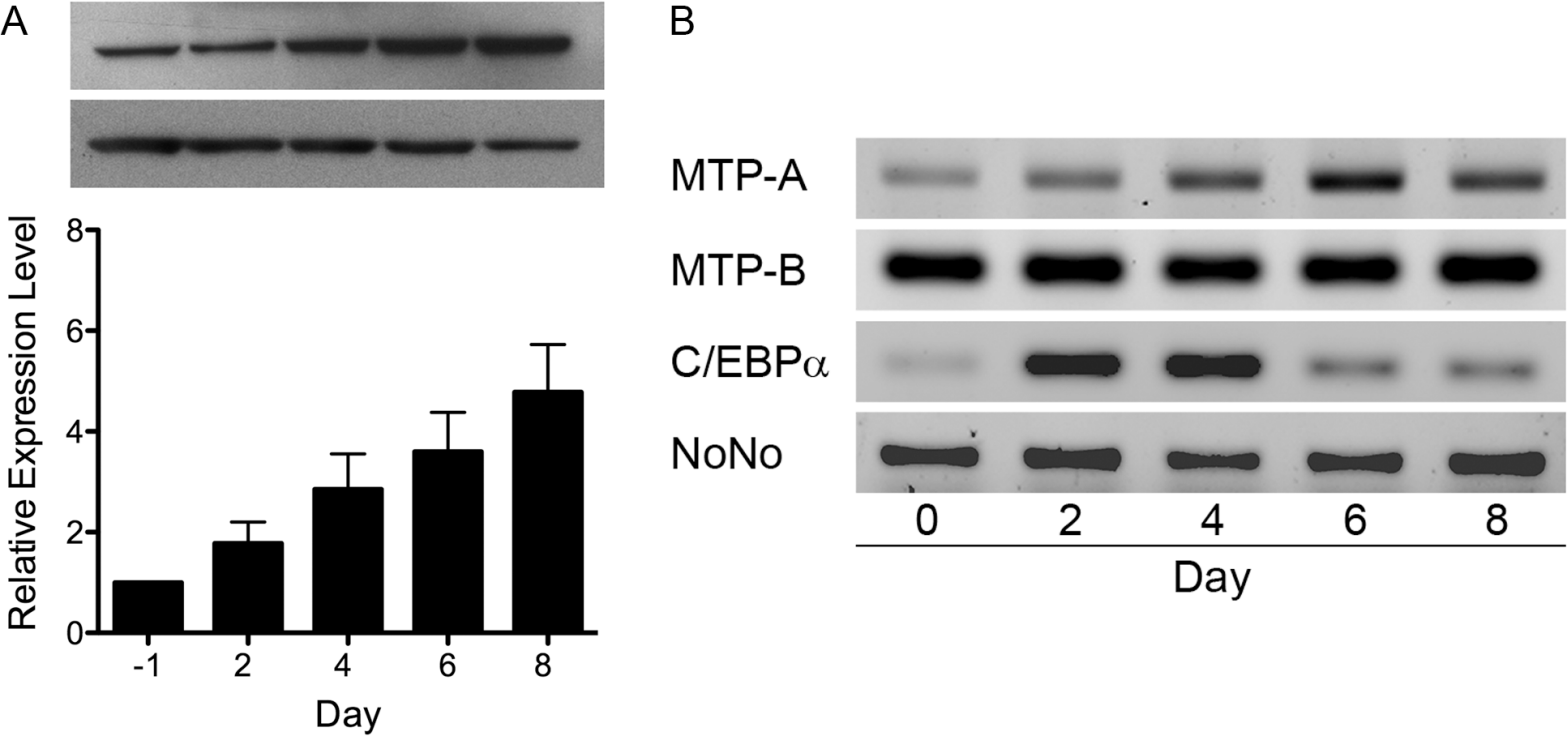
MTP protein and mRNA in differentiating 3T3-L1 cells. (A) 3T3-L1 cells were grown to confluence (day -1) and induced to differentiate on day 0. Equal amounts of cell lysate protein (20 μg), collected on the specified days, were separated by SDS-PAGE, blotted to nitrocellulose and probed for MTP. The relative amounts of MTP compared to day 0 were determined by densitometry. **Top:** representative immunoblots for MTP and β-actin. **Bottom:** Densitometric analyses (mean ± s.d.) from 3 separate experiments. (B) 3T3-L1 cells were grown to confluence and induced to differentiate (day 0). On the specified days, cells were recovered and total RNA isolated. RT-PCR was run using primers to amplify MTP-A, MTP-B, C/EBPα, and NoNo. NoNo was used as reference gene [24].

Previous studies in our laboratory demonstrated that MTP is expressed in 3T3-L1 cells and suggested that expression increases upon differentiation into adipocytes. To follow up on that observation, we grew 3T3-L1 cells to confluence (day -1), and induced them to differentiate on day 0. The cells were collected at different times during the differentiation process, and both protein and RNA were isolated. MTP protein expression, as monitored by immunoblot analysis, increased linearly over the 8-day period (Fig 1A), doubling by day 2 and increasing nearly fivefold by day 8. One-way ANOVA revealed a statistically significant difference in MTP levels as a function of differentiation time (*F*(4,10) = 5.819, p = .011). Tukey’s HSD post hoc test indicated that MTP levels at days 6 and 8 were greater than at day 0 (p<0.05) and levels at day 8 were greater than at day 2 (p<0.05). mRNA levels for MTP-A (F(4,10) = 2.042, p = 0.16, n = 3) and MTP-B (F(4,10) = 0.816; p = 0.54, n = 3) did not change significantly during differentiation (Fig 1B). MTP-A mRNA levels did seem to trend upward over the first 6 days; however, the levels were quite variable between experiments, due in part to the fact MTP-A mRNA levels are very low, requiring 35 PCR cycles for detection. As a control for cell differentiation we monitored the expres-sion of C/EBPα mRNA and demonstrated that it increased markedly during differentiation (Fig 1B).

### Effect of MTP inhibition on movement of triglyceride out of the cell

**Fig 2:**
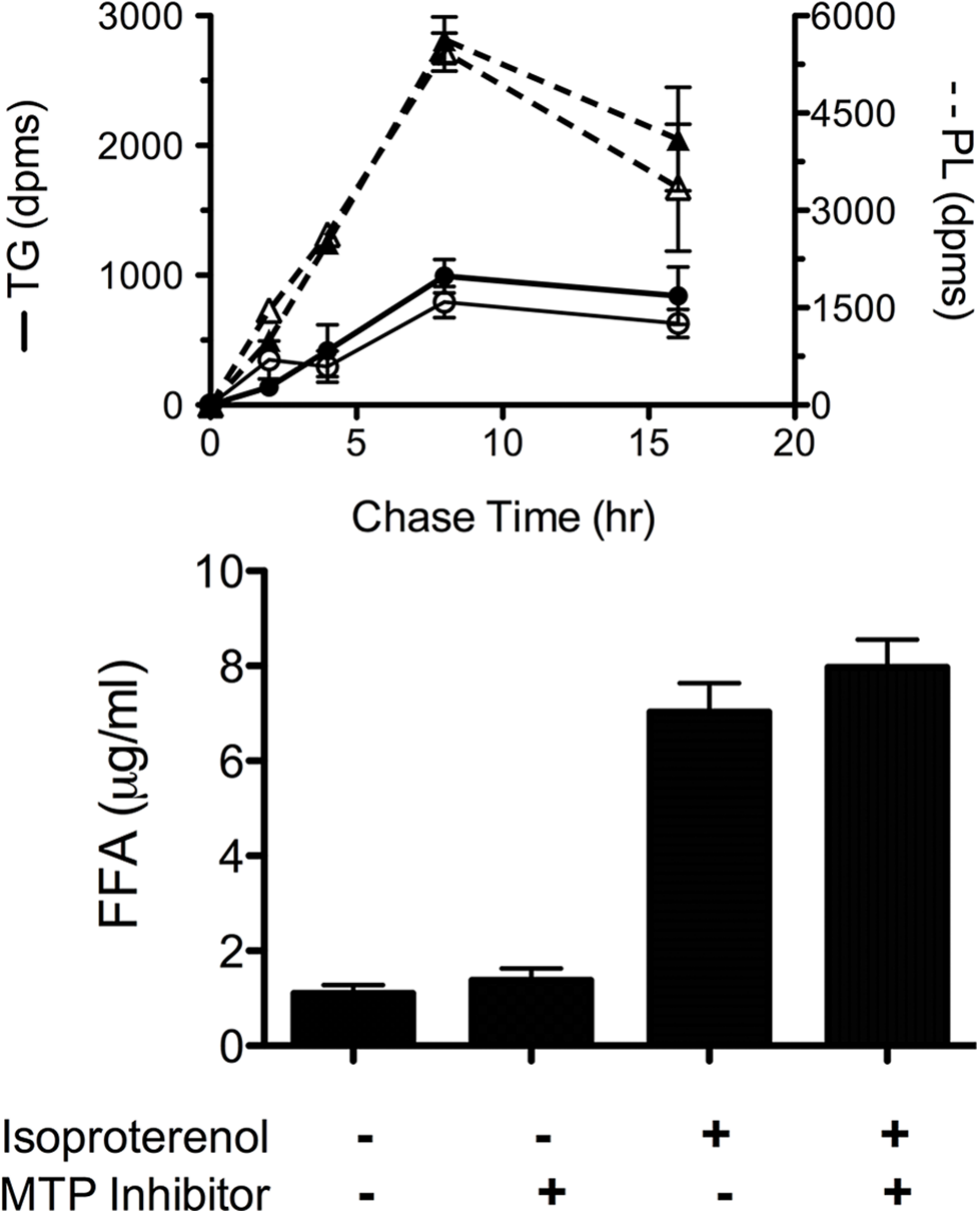
The effect of MTP inhibition on triglyceride (TG) and phospholipid (PL) secretion and lipolysis in adipocytes. (A) 3T3-L1 cells were induced to differentiate and on day 6, the cells were incubated with [^14^C]-acetate for 24 h. The media was removed, the cells washed, and fresh media was added with and without CP346086 (30 nM). The appearance of radioactivity in the TG and PL fractions was followed over 16 h. Solid symbols–control; open symbols–inhibitor. (B) Differentiated 3T3-L1 cells were incubated with MTP inhibitor (CP346086) for 45 min. The media was replaced with fresh media (DMEM, 2% fatty acid free BSA) with or without inhibitor and with or without isoproterenol (100 μM). After a 20 min incubation period, the media was removed and unesterified fatty acids were analyzed by gas chromatograph. For both experiments, data represent mean ± s.d., n = 3.

Increases in MTP protein with differentiation suggested to us that MTP might be involved in cellular triglyceride transport. Since the most widely characterized function of MTP is packaging lipid for secretion, we designed experiments to determine if differentiating 3T3-L1 cells secrete lipid in an MTP-dependent fashion. We utilized two approaches. In the first approach we induced 3T3-L1 cells to differentiate, and on day 6 we quantitated the mass of triglyceride and phospholipid accumulating in the media over a 24 hr period in the presence and absence of an MTP inhibitor (CP346086, 30 nM). The data were expressed as the ratio of the mass of lipid found in the presence of the inhibitor to that found in the absence of the inhibitor. The inhibitor had no effect on the mass of triglyceride (inh/cont = 0.92 ± 0.39; n = 6) or phospholipid (inh/cont = 1.11 ± 0.31; n = 7) recovered in the media. Likewise, we found no difference in the cellular phospholipids (356 ± 9 vs. 380 ± 28 μg, control vs. inhibitor, n = 3) or triglycerides (1235 ± 109 vs. 1130 ± 79 μg, control vs. inhibitor, n = 3). In the second approach, we incubated differentiated (day 6) 3T3-L1 cells with [^14^C]-acetate for 24 hr and measured the radioactivity appearing in the triglycerides and phospholipids in the media in the presence and absence of the MTP inhibitor (CP346086). The inhibitor had no effect on the amount of labeled lipid recovered in the media at any of the time points (Fig 2A). It is important to note that less than 1% of cellular triglyceride and less than 10% of cellular phospholipid was found in the media as measured by mass or radioactivity.

To determine if MTP was involved in the mobilization of fat from the cell via lipolysis, we quantitated fatty acid release from differentiated 3T3-L1 cells after stimulation with the β-adrenergic agonist, isoproterenol. Forty-five minutes prior to and during stimulation, the cells were exposed to MTP inhibitor (CP346086, 30 nM). The results are shown in Fig 2B. We found no difference in the mass of fatty acid released in the presence or absence of the inhibitor. Similar results were obtained when we used other inhibitors (BMS212122, BMS197636). The results indicate that the lipid transfer activity of MTP is not critical for the mobilization of fatty acids from adipocytes.

To determine if MTP activity was necessary for droplet formation or maturation, 3T3-L1 cells were grown to confluence and induced to differentiate in the presence and absence of MTP inhibitor (CP346086, 30 nM). The inhibitor had no effect on differentiation as assessed by the percent of cells that contained lipid droplets, nor was there any apparent effect on the number of droplets per cell (S1 Fig).

### Localization of MTP in basal and differentiated 3T3-L1 cells

**Fig 3:**
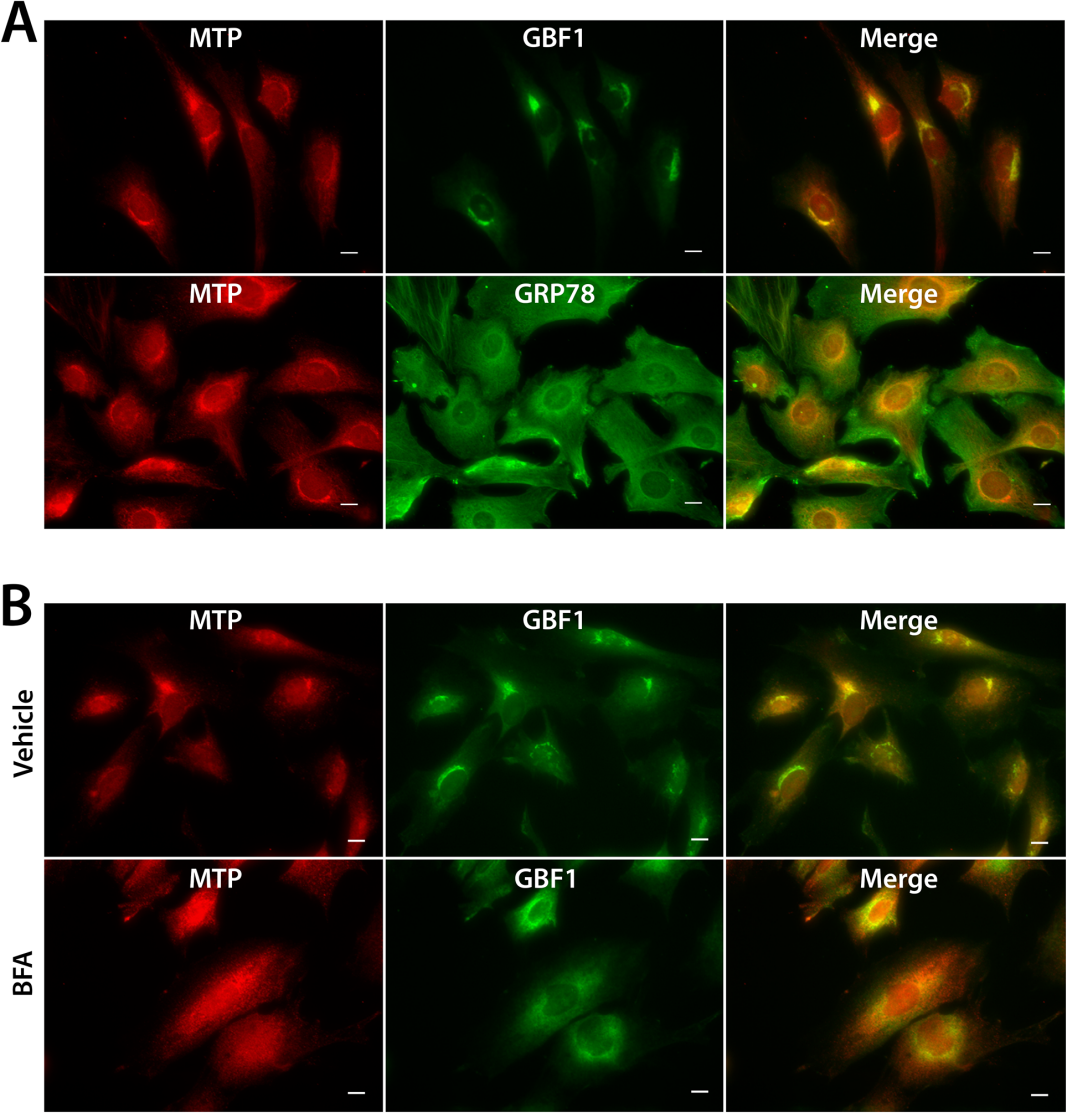
Cellular location of MTP in basal 3T3-L1 cells. (A) 3T3-L1 cells were grown in chamber slides and prior to reaching confluence, the cells were fixed and stained as indicated. MTP staining in the juxtanuclear region of the cells co-localizes with GBF1, but does not co-localize with the ER marker GRP78. Overlap of GRP78 and MTP was noted in the non-juxtanuclear region. (B) 3T3-L1 cells, plated as above, were treated with brefeldin A (5 μg/ml, 3 hr), fixed and stained for MTP and GBF1. Brefeldin A treatment leads to redistribution of MTP from the juxtanuclear region, similar to what occurs with GBF1. Bars = 10 μm.

Non-confluent, undifferentiated 3T3-L1 cells, grown in chamber slides were fixed and stained for MTP. In addition, 3T3-L1 cells were grown to confluence in chamber slides and induced to differentiate. On day 6 of differentiation, the cells were fixed and stained for MTP. As reported previously, in undifferentiated 3T3-L1 cells MTP was found primarily within the juxtanuclear regions of the cells [20] (Fig 3A), typical of Golgi staining. In fact, we found significant overlap of MTP with GBF1, an Arf guanine exchange factor that has been shown to localize with *cis* elements of the Golgi complex [28]. Brefeldin A treatment, which leads to disassembly of the Golgi complex and redistribution of GBF1 into the ER [28], also resulted in loss of juxtanuclear staining of MTP (Fig 3B). MTP was not solely localized within the juxtanuclear regions of the cell. Indeed, there is overlap of MTP staining with GRP78 (Fig 3A), suggesting that MTP is also present within the ER.

**Fig 4:**
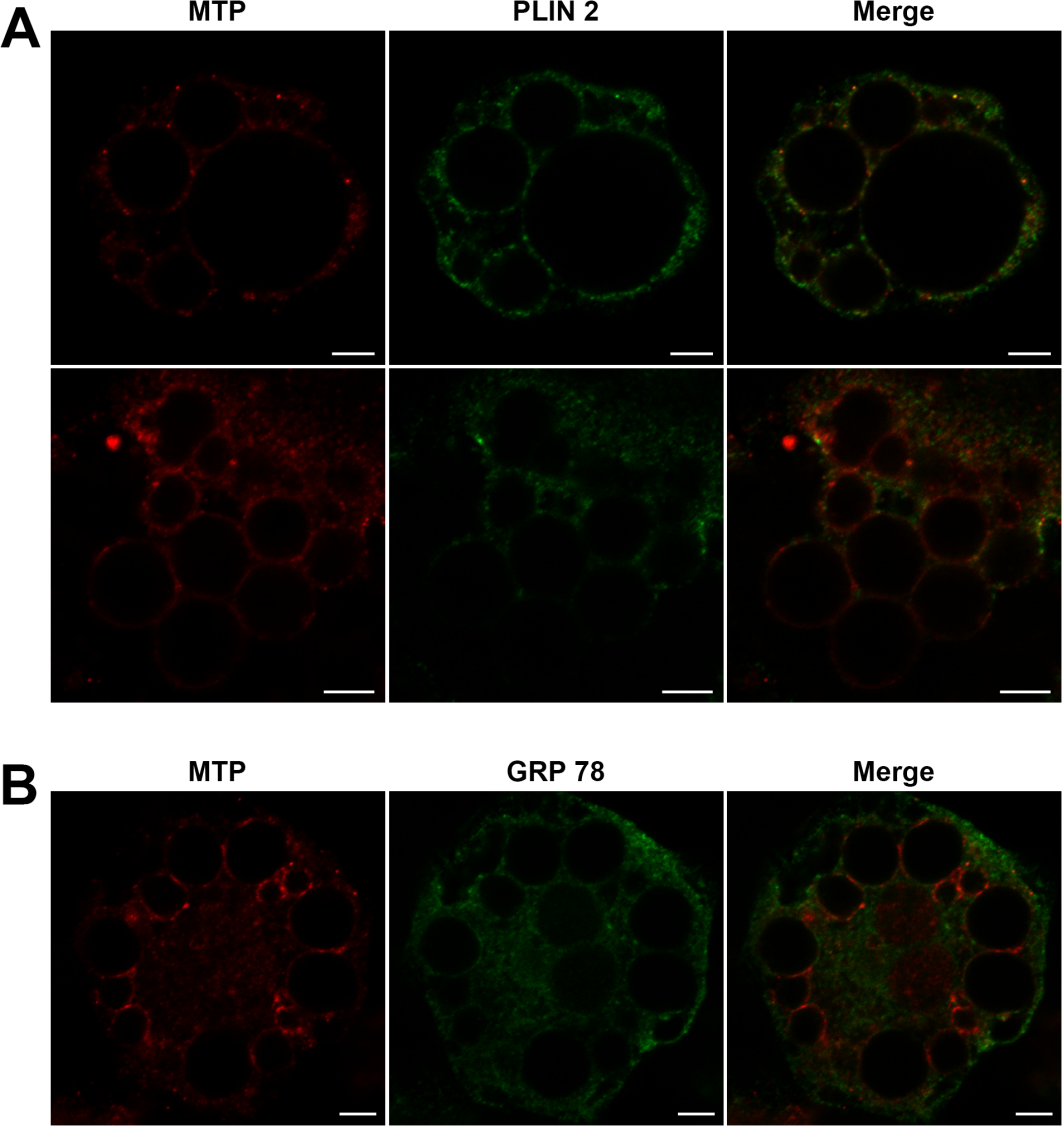
MTP location in differentiated 3T3-L1 cells. (A) 3T3-L1 cells were differentiated for 6 days, fixed and stained with antibodies to MTP (red) and perilipin (PLIN) 2 (green) and appropriate secondary antibodies and examined by confocal microscopy. MTP appears as puncta on the surface of lipid droplets. In some droplets the puncta are widely scattered; in others they appear to coat the droplet. MTP is not on all droplets. Perilipin 2 staining is very similar to that of MTP; however, there was little overlap (13%) of the MTP and perilipin 2 fluorescence. (B) Differentiated 3T3-L1 cells were stained with antibodies to MTP (red) and GRP78 (green) and examined by confocal microscopy. MTP puncta are clearly visible on the surface of the lipid droplets; however, the puncta do not substantially co-localize with the ER marker GRP78. Bars = 5 μm.

The pattern of MTP staining in differentiated cells (day 6) was quite different than undifferentiated cells with MTP appearing in a punctate pattern on the surface of lipid droplets throughout the cells (Fig 4A). Some droplets, especially the larger droplets, had few puncta widely scattered over the surface, whereas smaller droplets often showed several adjacent puncta, appearing as a “coat” on the surface. Some droplets did not appear to have MTP. To determine if the association of MTP with lipid droplets was similar to an accepted lipid droplet surface protein, we compared the pattern of MTP staining with that of perilipin 2 (Fig 4A, PLIN 2), as its pattern of association with lipid droplets in adipocytes seemed to parallel that of MTP better than any other protein in the perilipin family [29]. Interestingly, the staining pattern of perilipin 2 was similar to that of MTP. It appeared as scattered puncta on some droplets and as a “coat” on other droplets. Still other droplets did not appear to have perilipin 2. Qualitatively, there was less perilipin 2 staining on these droplets compared with MTP. We examined the overlap of MTP and perilipin 2 staining and found that only 13.0 ± 3.1% of the MTP fluorescence was within 0.2 μm of perilipin 2 fluorescence.

**Fig 5:**
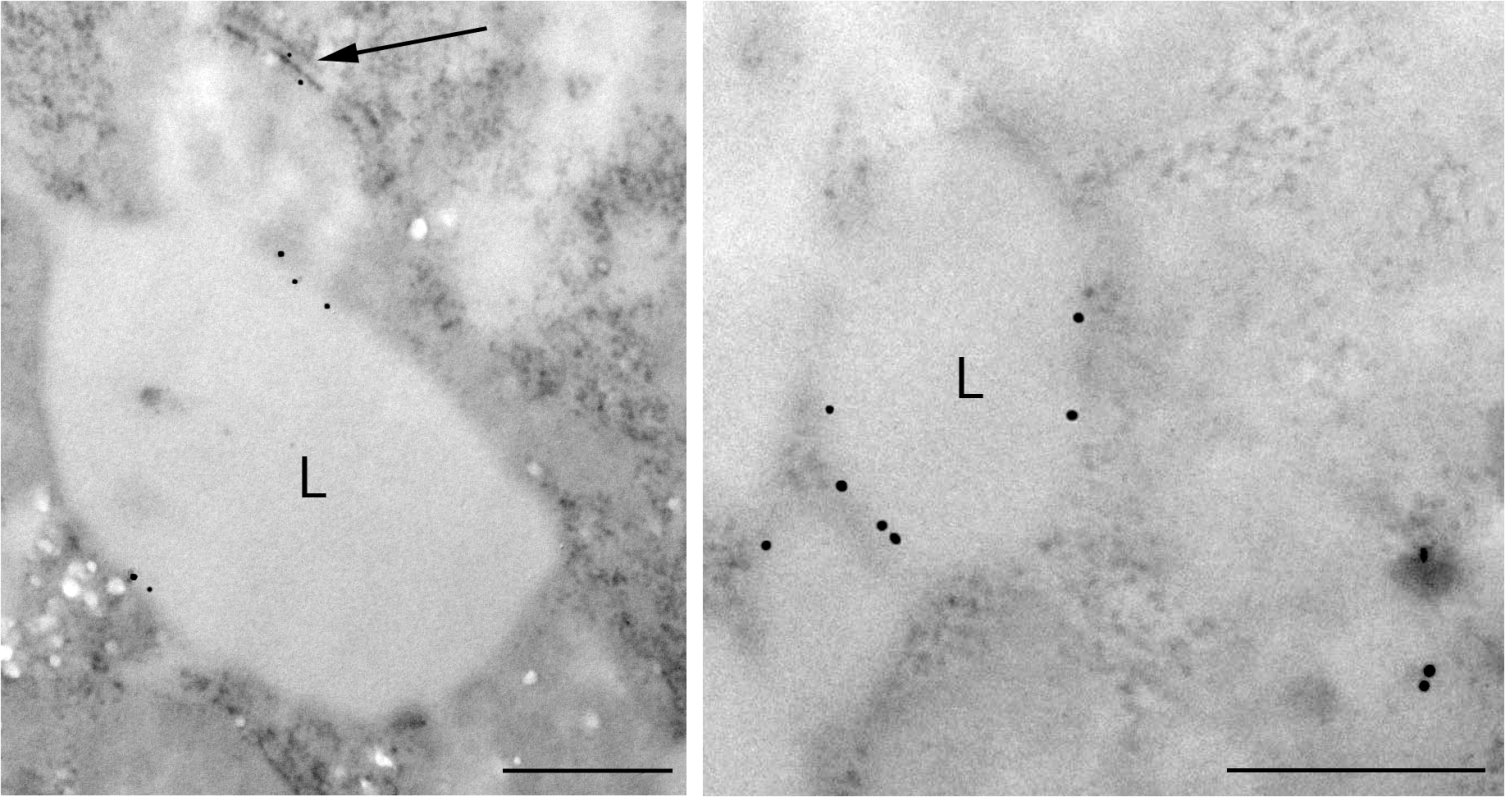
Microscopic studies demonstrating MTP on the surface of lipid droplets in 3T3-L1 cells. 3T3 cells were differentiated and MTP was visualized using MTP antibody followed by 10 nm gold conjugated secondary antibody. MTP staining was positive and cell specific. The staining appeared to be on the surface of the droplets and not associated with membranous structures associated with the droplet. Staining was observed with rough ER membranes as noted by the arrow. Nuclei were negative for immunogold labeling as were controls stained with immunogold without primary antibody. Bar = 500 nm

To explore the location of MTP with regard to the surface of the lipid droplet, we probed the overlap of MTP puncta on the droplets with the ER marker GRP78 using confocal microscopy (Fig 4B). Importantly, we did not observe overlap of the droplet puncta with the GRP 78, suggesting that droplet MTP was not part of the ER. To examine this further, we performed immunogold electron microscopy experiments on differentiated 3T3-L1 cells (Fig 5). Immunogold staining was positive and cell specific. Little to no staining was observed outside cells. Importantly, immunogold staining was prominent on the surface of lipid droplets (L), perhaps more prominent on smaller droplets (1 μ) and less prominent as the size of the droplet increased. Regardless of the size of the droplet, the colloidal gold staining around the droplet was not associated with membranous structures, but appeared to be on the surface of the droplet. MTP staining of a rough ER membrane separate from lipid droplets is clearly evident (arrow). The results provide strong evidence that MTP is indeed on the surface of the droplet.

### MTP is associated with isolated lipid droplets

**Fig 6:**
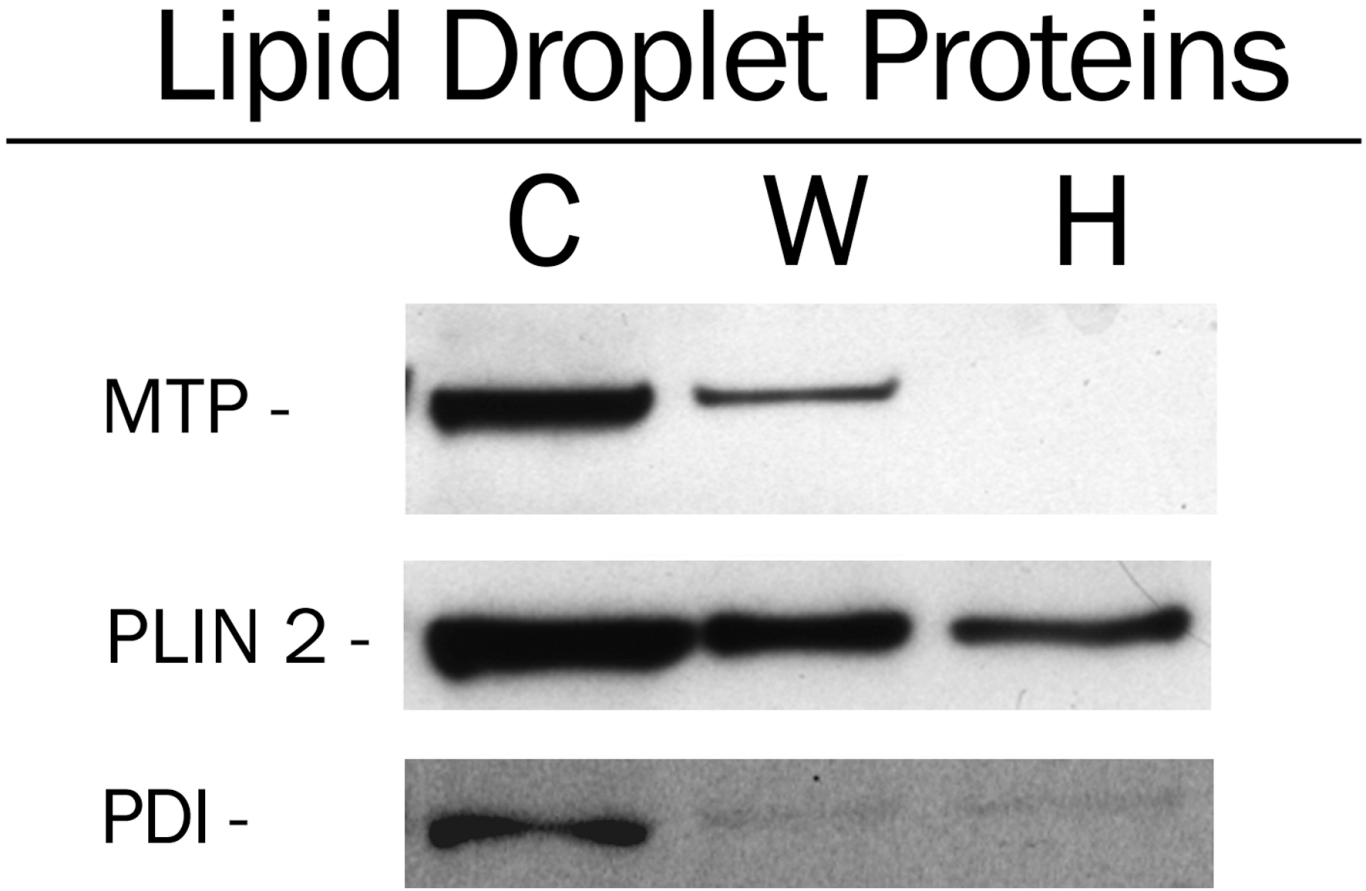
MTP is associated with isolated lipid droplets. 3T3-L1 cells were induced to differentiate and on day 6 lipid droplets were isolated as described in Experimental Procedures. Lipid droplets floating at the top of the discontinuous sucrose gradient were recovered (C, Control). Aliquots of this fraction were incubated with equal volumes of hypotonic lysis medium (Washed, W) or 0.2 M Na_2_CO_3_, pH 11.0, (Hi pH, H), re-floated through the discontinuous sucrose gradient, and recovered. All fractions were delipidated using -80°C acetone. The precipitated lipid droplet proteins were solubilized in SDS PAGE solubilizing buffer and analyzed by immunoblotting.

3T3-L1 cells were induced to differentiate, and on day 6 of differentiation lipid droplets were isolated from the cells as described in Materials and Methods. The initial lipid droplet fraction floating to the top of the discontinuous sucrose gradient contained MTP as well as perilipin 2 (PLIN 2) and PDI (C, Fig 6). Washing the fraction by reflotation through the discontinuous gradient resulted in a 72% loss of MTP and 48% loss of perilipin 2 (W, Fig 6) that could be accounted for in large part by loss of sample, as we only recovered approximately 50–60% of the triglyceride and phospholipid in the washed (W) lipid droplets. Incubation of the initial lipid fraction in 0.1 M sodium carbonate, pH 11, followed by reflotation resulted in near total loss of MTP (H, Fig 6) and a slight decrease in perilipin 2 compared with the washed (W) sample. PDI levels were reduced (66%) by reflotation (W) and high pH treatment (H). In fact, there was little difference in the PDI levels between the two samples, suggesting that MTP in the isolated lipid droplet fraction is not associated with contaminating ER membranes. The results are consistent with the microscopic studies suggesting that MTP is indeed associated with lipid droplets in 3T3-L1 cells.

### MTP localizes on hepatic lipid droplets

**Fig 7:**
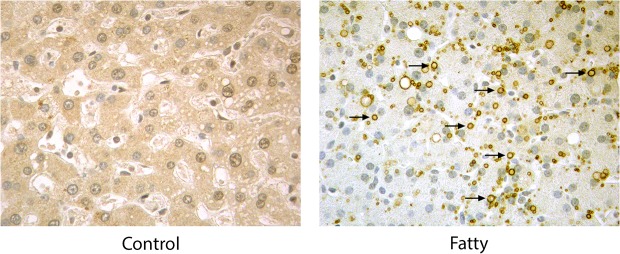
MTP associates with lipid droplets in “fatty” human liver. Sections of control and “fatty” human liver were stained with antibody to MTP and DAB-conjugated secondary antibody. Immunoreactive product was found throughout hepatocytes as previously reported for mouse liver [21], and it was especially prominent on lipid droplets (arrows).

Immunohistochemical studies were performed on control and fatty human liver to determine if MTP associated with lipid droplets in cells other than adipocytes. In control liver sections, MTP immunoreactive product was prominent in all hepatocytes, and the pattern of staining was consistent with localization in the ER (Fig 7). In fatty liver sections, MTP staining was visible throughout the hepatocytes, but was especially prominent on cytosolic lipid droplets (arrows), indicating that in a cell in which MTP functions in the assembly of lipoproteins within the lumen of the ER, it also associates with cytoplasmic lipid droplets.

## Discussion

Previous studies from our laboratory established the presence of MTP in adipose tissue as well as in the pre-adipocyte cell line, 3T3-L1 [20]. Our immunohistochemical studies with mouse tissues and our immunofluorescence studies with differentiated 3T3-L1 cells suggested that MTP was associated with lipid droplets; however, we were unable to determine if MTP was actually on the droplet surface or simply appeared to be on the surface as the droplets expanded within the cytoplasm. Furthermore, based on the apparent clustering of MTP around small lipid droplets in differentiated 3T3-L1 cells, we speculated that MTP might be participating in the accretion of lipid in droplet biogenesis [20]; however, we could not discount other possible roles for MTP in adipocytes. In the current studies we have used biochemical and microscopic approaches to probe more specifically the location of MTP within the cell, especially with regard to the lipid droplet surface. In addition, we have explored potential roles for MTP with regard to lipid droplet formation and the transport of triglyceride out of the cell, either as a lipoprotein particle or via lipolysis. Our studies demonstrate that (1) MTP levels increase robustly when 3T3-L1 cells are induced to differentiate, (2) Inhibition of MTP has no effect on differentiation nor is MTP activity required for lipid droplet formation, (3) MTP does not play a role in the secretion of triglyceride from 3T3-L1 adipocytes, nor does it play a role in the β-adrenergic stimulation of lipolysis; (4) MTP is localized primarily within the Golgi compartment of basal undifferentiated 3T3-L1; however (5) it is associated with the surface of lipid droplets in differentiated 3T3-L1 cells as well as with lipid droplets in human hepatocytes.

The marked increase in MTP levels as 3T3-L1 cells differentiate (Fig 1A), especially the twofold increase in the first two days, points to a role for MTP in the early stages of adipogenesis. Interestingly, MTP mRNA levels were basically unchanged over the 8-day differentiation period (Fig 1B), suggesting that the increase in protein occurs at the post-transcriptional level. It is important to note that Chang *et al*. [30] reported an increase in MTP protein levels but not MTP mRNA in livers from *Lep* ^*ob/ob*^ */ADFP* ^*-/-*^ mice compared with *Lep* ^*ob/ob*^ */ADFP* ^*+/+*^ mice. The increase in MTP was associated with increased VLDL secretion rates and decreased hepatic triglyceride content leading to an improvement in fatty liver grossly and microscopically. Thus, the increased MTP is a response that protects the liver from becoming steatotic [31]. We speculated that the increase in MTP in differentiated 3T3-L1 cells might reflect a cellular response to limit triglyceride accumulation. However, 3T3-L1 cells do not express apoB, but they do express apoE [32], and we questioned if it were possible for MTP to facilitate the formation of triglyceride-rich lipoproteins containing apoE, thereby reducing cellular triglyceride levels. Our results demonstrate that differentiated 3T3-L1 cells secrete very little triglyceride or phospholipid (Fig 2A). Furthermore, MTP inhibition has no effect on the amount of either lipid recovered in the cells or the media from these cells (Fig 2A). Likewise, MTP inhibition has no effect on β-adrenergic stimulation of lipolysis (Fig 2B). Thus, the increased expression of MTP protein during differentiation does not seem to be a response to limit cellular triglyceride accumulation by facilitating its movement out of the cell either as a lipid complex or via lipolysis.

Understanding the precise location of MTP within the cell and its movement during differentiation is critical to delineating its role in lipid droplet biology. Our microscopic studies show that MTP is prominent within the Golgi complex of basal 3T3-L1 cells. Additional microscopic studies (Figs 4 and 5) coupled with cell fractionation studies (Fig 6) on differentiated 3T3-L1 cells provide strong evidence that MTP is indeed associated with the surface of the droplet. Importantly, the pattern of MTP staining on lipid droplets is similar to that of perilipin 2 (ADRP), an authentic lipid droplet surface protein [33]. Indeed we found MTP and perilipin 2 on many of the same droplets (Fig 4A); however, there was little evidence for co-localization of the two proteins. This does not mean the two proteins could not work coordinately to influence lipid droplet biology. Furthermore, the association of MTP with lipid droplets is not restricted solely to adipocytes as we also found it associated with lipid droplets in human liver (Fig 7). MTP has long been thought to be localized to the endoplasmic reticulum; however, studies in our laboratory have shown MTP to also be present within the Golgi complex of hepatocytes [21], where it functions in lipoprotein assembly [34]. Its presence within the Golgi compartment of 3T3-L1 cells could prove important in explaining how MTP traffics to the lipid droplet. Indeed, recent evidence links lipid droplets to the secretory pathway, specifically the reported association of GBF1 with lipid droplets [35], and recent studies suggesting the involvement of ARF1/COPI machinery in lipid droplet genesis [36]. In addition, GBF1 is important in the formation of COPI vesicles [37], and COPI vesicles have been shown to be important in the targeting of ATGL and ADRP (perilipin 2) to lipid droplets [38, 39].

Although there have been numerous studies focused on delineating the process of lipid droplet formation and maturation, the precise mechanisms by which either occurs still remain undefined. According to the most widely accepted model, droplets form as a result of the accumulation of neutral lipid within the bilayer of the ER membrane (for reviews see [40–44]). As triglyceride accumulates and coalesces in the interior of the bilayer, the bilayer distends locally into a “lens” of triglyceride surrounded by a phospholipid monolayer. With continued triglyceride synthesis, the lens expands and ultimately “buds off” into the cytosol as a nascent lipid droplet. At least three models have been proposed to explain the growth of lipid droplets. In the first model, large droplets form via fusion of smaller droplets [45, 46]. While there is indirect evidence that might be consistent with this proposal, there is limited direct evidence to support the “fusion” hypothesis. A second model suggests that lipid droplets grow as a result of the synthesis of triglyceride at the droplet surface. This proposal is supported by the fact that DGAT2 associates with lipid droplets [47]. More direct evidence for the synthesis of triglyceride at the droplet was provided by studies of Xu and coworkers [48]. From studies with *c*. *elegans* they propose that FATP1, an acyl CoA synthetase, and DGAT2 work as a complex at the ER/lipid droplet interface to synthesize triglyceride, which is then deposited into the lipid droplet. More recently, the elegant studies of Wilfling *et al*. [49] demonstrated that “growing” (or expanding) lipid droplets contain the isozymes for each step in triglyceride synthesis and that some of these enzymes localize to the droplet via bridges between the ER and the droplet. A third model of expansion is the lipid exchange and transfer model in which droplets grow via the transfer of lipid from adjacent lipid droplets. Gong and coworkers [50] proposed that Fsp27, enriched at the contact sites of lipid droplets in 3T3-L1 adipocytes, mediates lipid transfer from smaller to larger lipid droplets. They speculated that the presence of Fsp27 might be important for recruiting “lipid transfer proteins” which would enhance the exchange and transfer of lipid. Interestingly, in a subsequent paper [51] they reported that perilipin 1 interacts with the CIDE-N domain of Fsp27, enhancing lipid exchange, transfer and growth. Their evidence suggested that cooperation between Fsp27 and perilipin 1 was necessary for efficient droplet growth.

Given the function of MTP as a triglyceride transfer protein, its ability to transfer lipid to protein as well as to facilitate the formation of lipid droplets in microsomes [52], and its presence on lipid droplets as shown in our studies, one can easily envision a role for MTP in any of the three models of lipid droplet expansion. For instance, Read *et al*. reported that helix A within MTP (amino acids 725–736) acts in a manner similar to viral fusion peptides, disrupting the phospholipid monolayer and providing access to triglyceride within the bilayer [53]. In a similar manner, MTP on the surface of lipid droplets might disrupt the phospholipid monolayer, facilitating the fusion of two droplets. However, the main function of MTP seems to be as a lipid transfer protein. It could provide substrate for triglyceride synthesis at the surface of the droplet or transfer newly synthesized triglyceride into the droplet or from one droplet to another. Studies are underway to explore these possibilities.

## Supporting Information

S1 Fig The Effect of MTP Inhibition on Droplet Formation in 3T3-L1 Cells. 3T3-L1 cells were grown to confluence and induced to differentiate (day 0) in the absence (A) and presence (B) of MTP inhibitor (CP 346086, 30 nM). The media was removed each day, and fresh media (with and without inhibitor) was added. On day 4 the cells were fixed, and images were captured randomly and in an unbiased fashion using differential interference contrast (DIC) microscopy. The histogram (C) presents the percent of cells that contained lipid droplets (n = 3 experiments). At least 8 images were analyzed per condition per experiment (≈1400 cells total per condition). Magnification = 425X.
(TIF)

S2 Fig Original blots for MTP and for β-actin for Fig 1A. (TIF)

S3 Fig Original blots for MTP, ADRP (perilipin 2), and PDI for Fig 6. (TIF)
