# Supplementary figures and images for: Microsomal Triglyceride Transfer Protein (MTP) Associates with Cytosolic Lipid Droplets in 3T3-L1 Adipocytes
Source: PLoS One. 2015 Aug 12;10(8):e0135598. doi: 10.1371/journal.pone.0135598 (PMC4534446; doi:10.1371/journal.pone.0135598)

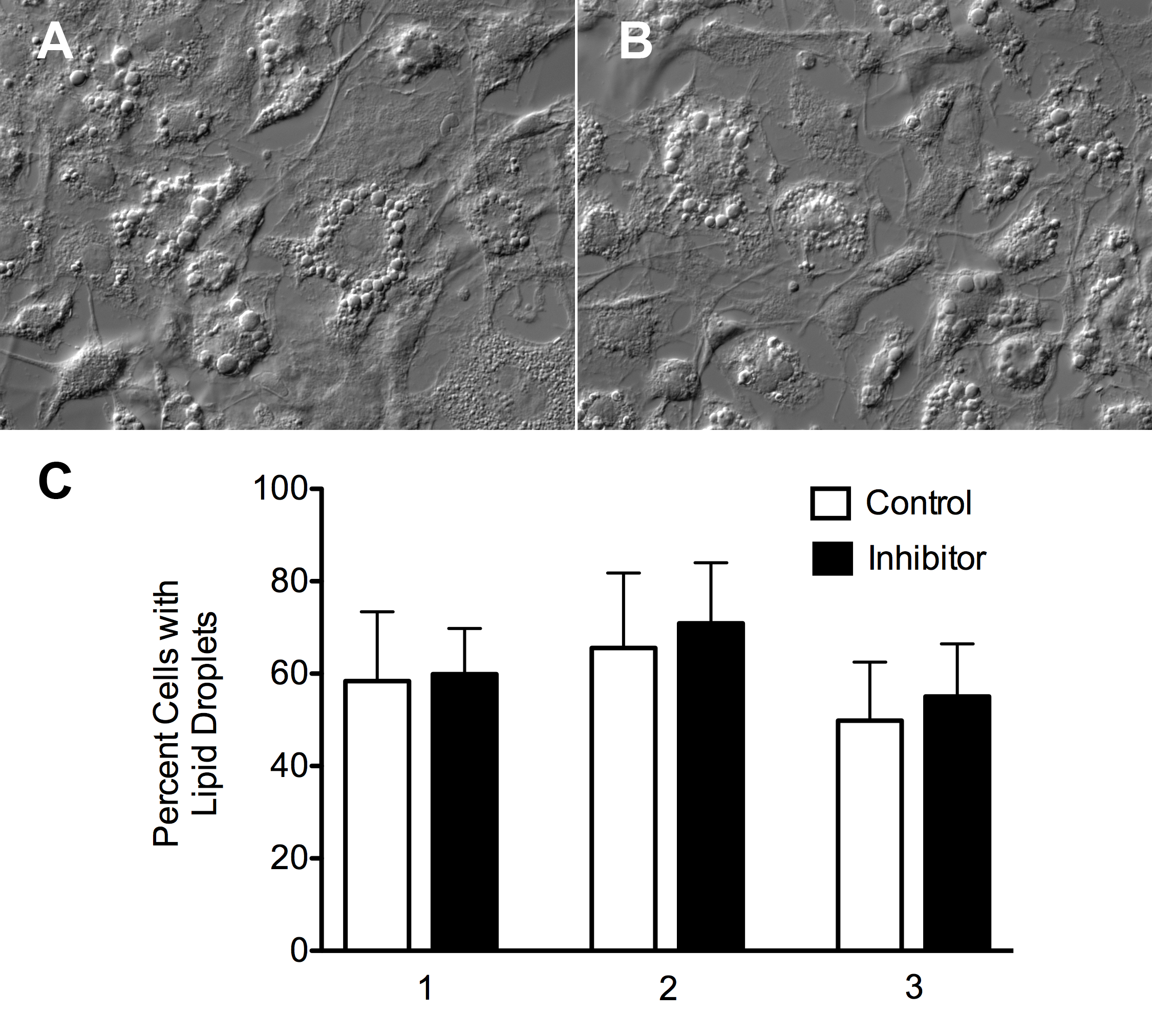

Supplement: S1 Fig — 3T3-L1 cells were grown to confluence and induced to differentiate (day 0) in the absence (A) and presence (B) of MTP inhibitor (CP 346086, 30 nM). The media was removed each day, and fresh media (with and without inhibitor) was added. On day 4 the cells were fixed, and images were captured randomly and in an unbiased fashion using differential interference contrast (DIC) microscopy. The histogram (C) presents the percent of cells that contained lipid droplets (n = 3 experiments). At least 8 images were analyzed per condition per experiment (≈1400 cells total per condition). Magnification = 425X. (TIF) [file pone.0135598.s001.tif]

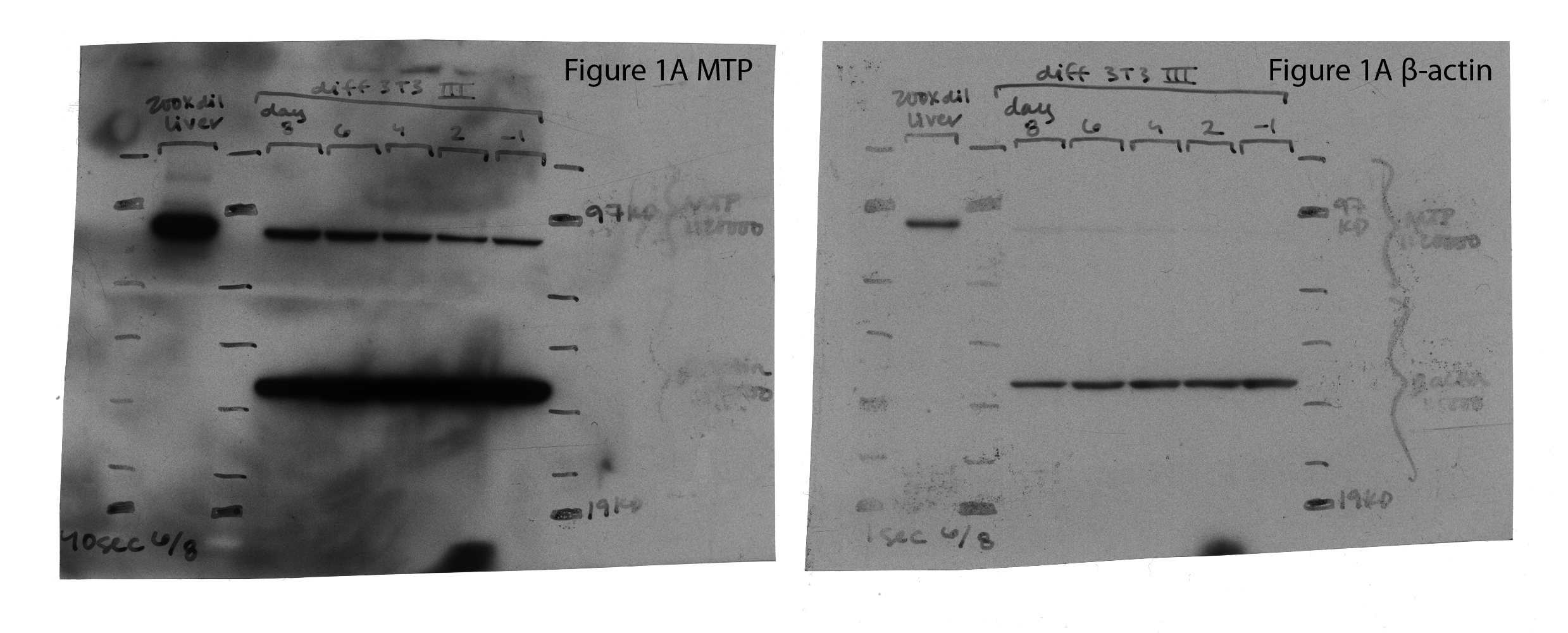

Supplement: S2 Fig — (TIF) [file pone.0135598.s002.tif]

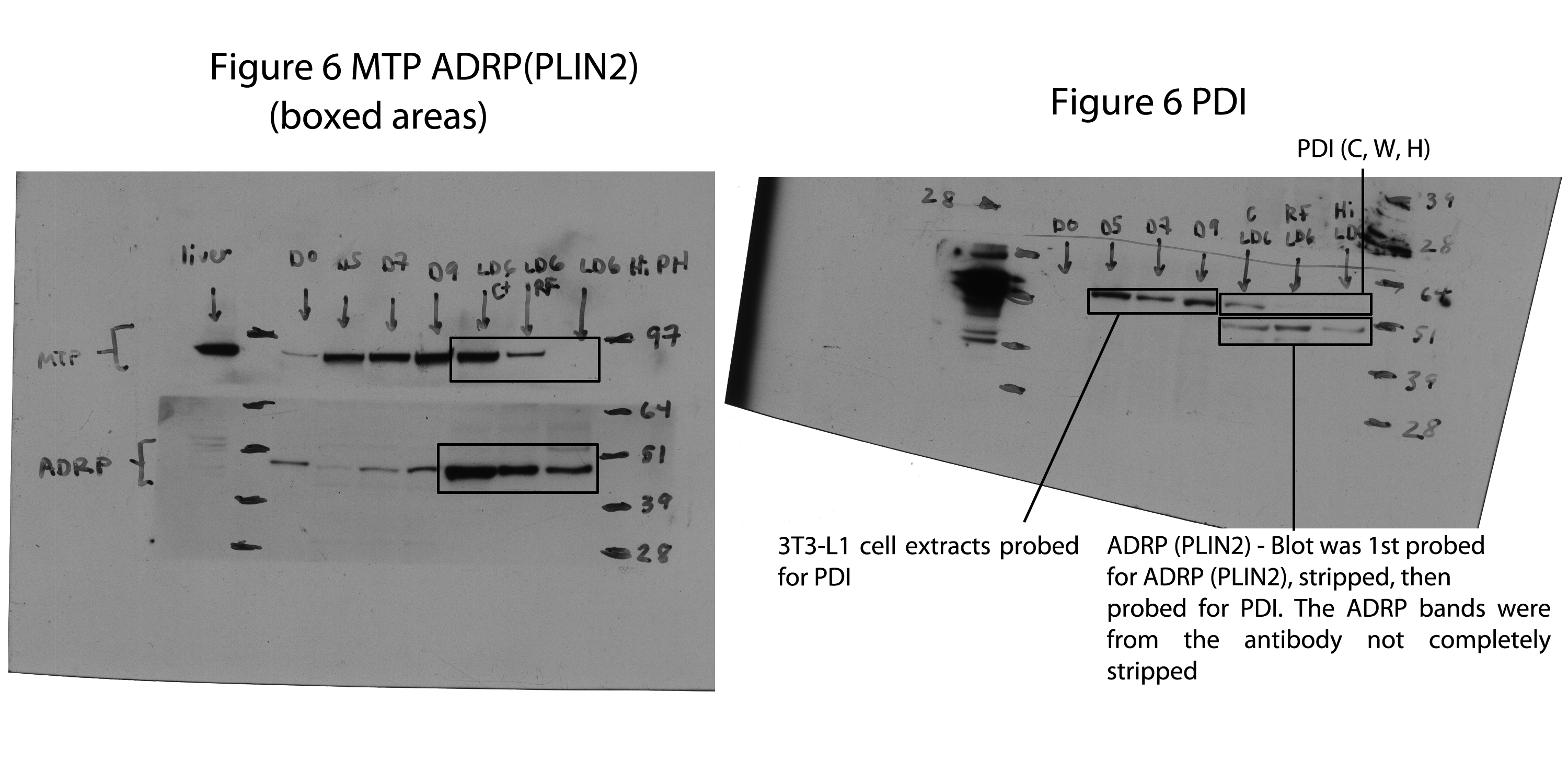

Supplement: S3 Fig — (TIF) [file pone.0135598.s003.tif]
